# Interactive Narrative–Based Digital Health Interventions for Vaccine Communication: Protocol for a Scoping Review

**DOI:** 10.2196/51137

**Published:** 2024-02-09

**Authors:** Ahmed Haji Said, Kate Winskell, Robert A Bednarczyk, Erin E Reardon, Lavanya Vasudevan

**Affiliations:** 1 Hubert Department of Global Health Rollins School of Public Health Emory University Atlanta, GA United States; 2 Woodruff Health Sciences Center Library Emory University Atlanta, GA United States

**Keywords:** narrative, storytelling, digital health, social media, interactive, vaccine, vaccination, vaccine hesitancy, vaccine communication

## Abstract

**Background:**

Interactive narrative–based digital health interventions hold promise for effectively addressing the complex determinants of vaccine hesitancy and promoting effective communication across a wide range of settings and vaccine types. Synthesizing evidence related to the implementation and evaluation of these interventions could offer valuable perspectives for shaping future strategies in vaccine communication. Prior systematic and scoping reviews have examined narrative-based vaccine communication interventions but not the inclusion of interactivity in such interventions.

**Objective:**

The overall objective of the scoping review is to summarize the evidence on the use of interactive narrative–based digital health interventions for vaccine communication. Specific research questions focus on describing the use of interactive narrative–based digital health interventions (RQ1), describing evaluations of the impact of interactive narrative–based digital health interventions on promoting vaccine uptake (RQ2), and factors associated with their implementation (RQ3).

**Methods:**

A detailed search string will be used to search the following databases for records that are relevant to the review questions: PubMed, Embase, Scopus, Web of Science, CINAHL, and PsycINFO. Two reviewers will independently screen the titles and abstracts of identified records against the predefined eligibility criteria. Subsequently, eligible records will undergo comprehensive full-text screening by 2 independent reviewers to assess their relevance to review questions. A data charting tool will be developed and used to extract relevant information from the included articles. The extracted information will be analyzed following the review questions and presented as a narrative summary. Tabular or graphical representations will be used to display review findings, as relevant.

**Results:**

Public health informationists were consulted to develop the detailed search strategy. The final search string comprised terms related to narrative communication, digital health, and vaccines. The search string was customized to each proposed publication database and implemented on April 18, 2023. A total of 4474 unique records were identified using the search strategy and imported into the Covidence (Veritas Health Innovation Ltd) review management software for title and abstract screening. Title and abstract screening of identified records are ongoing as of December 29, 2023.

**Conclusions:**

To our knowledge, this will be the first scoping review to investigate the features of interactive narrative–based digital health interventions and their role in vaccine communication. The goal of this study is to provide a comprehensive overview of the current research landscape and identify prevailing gaps in knowledge. The findings will provide insights for future research and development of novel applications of interactive narrative–based digital health vaccine communication interventions.

**International Registered Report Identifier (IRRID):**

DERR1-10.2196/51137

## Introduction

### Background

Vaccinations prevent more than 20 infectious diseases and avert 4-5 million deaths across the lifespan globally each year [1]. Yet, global progress on vaccinations has stalled in the last decade because of factors associated with vaccine complacency, convenience, or confidence [2]. These factors can result in vaccine hesitancy, a state of decisional ambivalence that manifests as a “delay in acceptance or refusal of vaccination despite availability of vaccination services” [2]*.* Vaccine hesitancy is blamed for backslides in vaccination coverage in high-income countries (eg, in Europe and the Americas), where issues with vaccine availability are less of a factor in suboptimal vaccine uptake [3]. Even in non–high-income settings, vaccine hesitancy may be present because of vaccination service interruptions and barriers to vaccine access [4]. The World Health Organization Strategic Advisory Group of Experts on Immunization noted that “vaccine hesitancy is complex and context-specific, varying with time, population, geographical location, and vaccine type” [2]. Passive or sloganized approaches frequently used by vaccination programs are insufficient to address the complexity and variability of the factors underlying vaccine hesitancy [5]. In a systematic review, Jarrett et al [6] found that dialogue-based communication strategies such as those leveraging mass media, social mobilization, and community influencers were effective in addressing vaccine hesitancy, whereas passive strategies such as those using posters and websites had a lower impact. One way to incorporate dialogue-based content may be through the use of narratives. The growth in the use of narratives for vaccine communication in recent years presents an opportunity to further study and innovate on its use to support vaccination uptake.

Narratives are ubiquitous in human society and have been used for persuasive health communication for decades [7,8]. Narrative-based communication strategies may be effective for understanding and addressing the complex determinants of vaccine hesitancy and enabling dialogue-based vaccine communication across varied settings and types of vaccines. The proliferation of digital technologies such as mobile phones has led to rapid and widespread sharing of health-related narratives via digital media (eg, social media platforms). These digital modalities of information communication may permit new ways for individuals to interact with narratives. For instance, interactive narrative–based digital health interventions, such as choose-your-own-adventure games, may offer individuals the chance to make decisions for their characters, influencing the unfolding of divergent paths and outcomes of their journeys [9]. Such interventions show a promising approach to storytelling by fostering self-efficacy that could be leveraged for vaccination promotion [10].

While prior systematic and scoping reviews have examined narrative-based communication for health [11,12], or specifically vaccines [13], the interactivity of narrative-based digital health interventions was not the focus. A synthesis of ways in which interactive narrative–based digital health interventions have been used for vaccine communication can facilitate the development and adaptation of novel applications to support vaccination. The goal of this scoping review is to summarize the evidence on the use of interactive narrative–based digital health interventions for vaccine communication.

### Concepts Included in This Review

#### Overview

The 4 key concepts included in this review are narrative communication, digital health, interactivity, and vaccine communication.

#### Narrative Communication

For the purposes of this review, we follow Hinyard and Kreuter [7] definition of narratives as: “any cohesive and coherent story with an identifiable beginning, middle, and end that provides information about scene, characters, and conflict; raises unanswered questions or unresolved conflict; and provides resolution.”

Narratives may be presented in different ways (eg, entertainment education, case histories, and testimonials) or delivered through different modalities (eg, social media, comics, and plays) [7,8]. The content of narratives may include “official stories that are constructed to tell an innocuous version of events or the position of a group, invented stories that are made up or fictional, firsthand experiential stories, secondhand stories (ie, retelling of someone else’s story), and culturally common stories that are generalized and pervasive in a cultural context” [7,14]. For this review, we are interested in describing all elements of narratives used in vaccine communication, including the use of characters, settings, plots, points of view, features, and themes.

#### Digital Health

Digital health is defined as “the use of information and communications technology in support of health and health-related fields” [15]. The phrase digital health encompasses computer-based (electronic health or “eHealth”) and mobile phone–based approaches for communicating health information and delivering health services. It also includes newer information and communication technology domains such as artificial intelligence [15]. Individuals may leverage digital technologies to cultivate social connections and access medical advice, including information on vaccines [10,16]. For this review, we are interested in describing all elements of digital health interventions that deliver narratives on vaccines, including the types of devices, modalities, and specific digital strategies as defined in the World Health Organization classification of digital health interventions [17].

#### Interactivity

For the purpose of this review, we define the term “interactive” as the active engagement of individuals with the narrative via digital health, aiming to raise awareness, empower behavior change, and ultimately lead to improved vaccination outcomes. Interactivity in narrative-based digital health interventions may allow individuals to make sense of stories related to vaccines and vaccination. Interactivity may take various forms depending on the specific narrative, digital medium, or platform being used. For instance, interactive narratives may transcend the boundaries of passive information consumption, empowering individuals to actively shape the narrative’s trajectory through decision-making processes (eg, games for health) [9], or individuals may respond to actions and outcomes of the narrative’s characters (eg, via likes or comments on posts), or feature in the narratives themselves (eg, as avatars). For this review, we are interested in describing ways in which interaction with narratives has been incorporated via digital health in vaccination communication interventions.

#### Vaccine Communication

For this review, we describe vaccine communication very broadly as any communication related to vaccines. The purpose of the communication could vary (eg, for mass communication to promote new vaccines, provider-patient communication, to reduce misinformation, counter vaccine hesitancy, or train health workers). Communication about vaccines can involve a wide range of mechanisms including improving knowledge or awareness, shaping attitudes and beliefs, and so forth. We are interested in describing different vaccine communication use cases where interactive narrative–based digital health interventions have been used. We will include all vaccine types and populations targeted for communication. The communication process will be examined for 5 key components: sender, channel, message, receiver, and feedback [18].

### Description of the Intervention

We are interested in any digital health interventions that incorporate narratives and interactivity for vaccine communication. We are interested in all types of narrative communication including games for health, entertainment education, storytelling, testimonials, and case histories. The entities delivering the narratives (ie, the messengers) may be formally trained (eg, doctors, public health experts, and researchers), laypersons (eg, community health workers, and peers), organizations, or health systems. The recipients may be persons receiving information on vaccinations or persons providing vaccination services.

The narrative vaccination communication process can take place entirely through a digital medium or via hybrid approaches that have at least 1 digital health component. For instance, in a hybrid approach, a provider may use a digital health aid to communicate vaccination narratives (eg, a narrative vaccination video on a tablet device) but follow up with a discussion (ie, interaction) about the video.

### Research Objectives

This scoping review aims to answer the following specific research questions (RQs):

RQ1: How have interactive narrative–based digital health interventions been used for vaccine communication?

RQ2: How have interactive narrative–based digital health interventions been evaluated for promoting vaccine uptake?

RQ3: What implementation factors are associated with the use of interactive narrative–based digital health interventions for vaccine communication?

## Methods

### Overview

The methods of this scoping review are reported in accordance with the PRISMA-ScR (Preferred Reporting Items for Systematic Reviews and Meta-Analyses extension for Scoping Reviews) checklist (Multimedia Appendix 1) [19].

### Inclusion Criteria

Due to the hierarchical nature of the research questions, all included articles need to fulfill the inclusion criteria for RQ1. Within this subset of research articles, a subset of articles will also satisfy the inclusion criteria for RQ2 and RQ3.

RQ1: How have interactive narrative–based digital health interventions been used for vaccine communication?

- Studies encompassing any form of vaccine communication irrespective of vaccine type. We will consider studies incorporating vaccine communication alongside other interventions (eg, nutrition)
- Studies using narrative communication where the target audience can engage with the narrative
- Studies where the narrative is delivered via digital health devices (eg, via mobile phones and tablets) and modalities (eg, SMS text messages, applications, and games for health), including hybrid approaches where at least 1 component is delivered digitally
- Studies published as original research articles, presenting empirical findings obtained from data collection efforts.

RQ2: How have interactive narrative–based digital health interventions been evaluated for promoting vaccine uptake?

- Studies that meet the criteria for RQ1
- Studies that have been evaluated for vaccine uptake (eg, whether individuals received a vaccination or not after exposure to the narrative intervention) or vaccination intention.

RQ3: What implementation factors are associated with the use of narrative-based digital health interventions for vaccine communication?

- Studies meeting the criteria for RQ1
- Studies reporting implementation factors (ie, barriers and facilitators) related to the implementation of interactive narrative–based digital health interventions for vaccine communication
- Studies reporting implementation outcomes (eg, feasibility, acceptability, adoption, cost, reach, usability, and sustainability) derived from the evaluation of interactive narrative–based digital health interventions for vaccine communication.

### Exclusion Criteria

We will exclude (1) publications classified as gray literature, protocols, trial registries, editorials, opinion pieces, and systematic and scoping reviews (ie, not an original research article); (2) original research articles for which full text are not available; (3) studies published in languages other than English for which certified translations in English are not available from the original source; (4) for RQ2, studies solely reporting intermediate outcomes related to vaccination knowledge, attitudes, or beliefs without data on vaccination intention or uptake; and (5) for RQ3 specifically, studies lacking empirical data on implementation factors or outcomes.

### Search Strategy and Data Extraction

Two public health informationists (ER and HR) were consulted to develop the search strategy. A detailed search string was formulated in PubMed (Textbox 1) using a combination of MeSH (Medical Subject Headings) terms and relevant keywords to cover the domains of narratives, digital health, and vaccine communication.

The following other databases were searched using customized versions of the search string presented in Textbox 1: PubMed, Embase, Scopus, Web of Science, CINAHL, and PsycINFO. Studies that were published since 2010 will be included in the review due to the proliferation of digital storytelling as a tool for health care delivery around that time [20]. The research articles will be limited to English only. Screening and data extraction will be conducted using Covidence review management software as follows:

- Titles and abstracts will be independently reviewed by 2 researchers and records will be sorted as included or excluded for the next stage of review based on consensus voting. Full-text screening of included abstracts and titles will be completed by 2 reviewers, and a similar consensus voting approach will be followed. A third independent researcher will be consulted if the reviewers do not reach a consensus, and the majority vote will apply.
- We will develop and implement a data extraction template in Covidence based on the inclusion criteria, research objective, and RQs. Characteristics of the studies (eg, date, author, research question, and study findings) will be documented using the template by 2 researchers for each included study.

During the data extraction process (Textbox 2), we will focus on gathering the following information from included studies.

**Search string used for identifying relevant records in PubMed.**

((“Narration”[Mesh] or Communication[Mesh]) or (narrat*[tw] or storytell*[tw] or story-tell*[tw] or “story telling”[tw] or storyline*[tw] or story[tw] or stories[tw] or conversation*[tw] or testimoni*[tw])) AND ((“Social Media”[Mesh] or “Mobile Applications”[Mesh] or “Smartphone”[Mesh] or “Telemedicine”[Mesh] or “Artificial Intelligence”[Mesh] or “Digital Technology”[Mesh] or “Computers”[Mesh]) or (digital[tw] or app[tw] or apps[tw] or “social media”[tw] or twitter[tw] or facebook[tw] or tiktok[tw] or instagram[tw] or Weibo[tw] or youtube[tw] or telegram[tw] or Whatsapp[tw] or Tumblr[tw] or Pinterest[tw] or snapchat[tw] or wechat[tw] or reddit[tw] or myspace[tw] or computer*[tw] or smartphone*[tw] or chat[tw] or blog*[tw] or game[tw] or gaming[tw] or games[tw] or gamification[tw] or weblog*[tw] or online[tw] or web-based[tw] or electronic[tw] or ehealth[tw] or e-health[tw] or “electronic health”[tw] or mhealth[tw] or m-health[tw] or “mobile health”[tw] or “artificial intelligence”[tw] or ai[tw] or “machine learning”[tw] or “deep learning”[tw] or chatbot[tw] or “chat bot”[tw] or chatgpt[tw])) AND ((“Vaccines”[Mesh] or “Vaccination”[Mesh]) or (vaccin*[tw] or immuniz*[tw] or immunis*[tw] or anti-vax*[tw] or antivax*[tw] or “anti vax”[tw] or anti-vaccin*[tw])) AND (English[Filter]

**Proposed elements of data extraction by review question.**

Study information: In a table of included studies, we will summarize study dates, publication information, study setting, participants, study designs, and objectives.

RQ1: We will summarize different elements involved in narrative communication across a range of contexts. Our analysis will include how individuals engage with interactive digital health interventions or modalities, examining the various modes of interaction and their impact on the overall narrative experience. The outcome of the narratives, examining factors such as their congruence with individuals’ personal values, their memorability, perceived realism, and other relevant elements will be described. Theories and frameworks used in the included studies such as Schank and Berman categorization of narratives (official, invented, first-hand experiential, second-hand, and culturally common), or other pertinent theoretical frameworks will be analyzed [14]. Furthermore, we will provide insights into the available evidence by vaccine type, whether the emphasis is solely on vaccination, and whether the vaccines are integrated with other health interventions.

RQ2: All elements described for RQ1, and evidence of impact on the uptake of vaccines, specifically examining whether individuals were vaccinated or not after being exposed to interactive narrative-based digital health interventions for vaccine communication. We will also review studies that have evaluated vaccination intention, which relates to individuals’ expressed willingness or plans to get vaccinated.

RQ3: We will include all elements described in RQ1, along with the implementation outcomes related to the use of narrative-based digital health interventions for vaccine communication. These implementation outcomes include outcomes such as acceptability, adoption, cost, reach, usability, sustainability, and others. Additionally, we aim to identify studies that report on the barriers and facilitators associated with implementing interactive narrative-based digital health interventions for vaccine communication. By examining these factors, we seek to gain a comprehensive understanding of the feasibility and implementation of utilizing such interventions for vaccine communication efforts.

### Data Analysis

Review findings will be communicated through a PRISMA (Preferred Reporting Items for Systematic Reviews) flowchart, tables, figures containing descriptive statistics, and narrative summaries corresponding to our research questions. Research findings will be published in a peer-reviewed academic journal and presented in scientific conference presentations in the forthcoming months.

### Ethical Considerations

Since the review used published journal articles exclusively, an ethical board review board was not necessary to conduct this review.

## Results

The detailed search string was implemented in the proposed databases searching for publications from inception until April 18, 2023. The search results (n=6836 records) were imported into EndNote Software (version 20; Clarivate) for the removal of duplicate records, and deduplicated records (n=4676) were uploaded to Covidence review management software for eligibility screening. Title and abstract screening is ongoing as of December 29, 2023. We anticipate the scoping review findings to be published in 2024.

## Discussion

### Principal Findings

Prior systematic and scoping reviews in the realm of vaccine communication have evaluated various interventions such as behavioral nudges [13] and dialogue-based communication interventions [21]. However, none of these reviews have specifically evaluated the effects of interactive narrative–based digital health interventions for vaccine communication. This scoping review aims to identify and synthesize relevant studies that describe interactive narrative–based digital health interventions tailored specifically for vaccine communication. Our primary goal is to synthesize empirical evidence on the ways these interventions are used, implemented, and evaluated and highlight gaps to inform future research and implementation.

### Implications for Research and Practice

The study findings may help researchers and public health practitioners understand how interactive narrative–based digital health interventions have been used to promote vaccinations and add context or perspective that may be missing from traditional messaging campaigns [2,5]. The findings from the scoping review may guide the future development of interactive narrative–based digital health interventions for vaccine communication and identify interventions for further evidence synthesis. Additionally, the review may highlight implementation outcomes linked to these interventions or successful components within them. Through a comprehensive synthesis of existing literature, the review may reveal effective strategies, challenges, and gaps in previously developed interventions. Furthermore, the review has the potential to stimulate new research questions or hypotheses aimed at addressing these gaps, thereby contributing to the dynamic landscape of vaccine communication.

Narratives are often exploited in the spread of vaccine misinformation. While the proliferation of digital devices such as mobile phones and tablets has enabled widespread reach of information through communication channels such as social media, they have also helped the spread of misinformation, thereby affecting the public’s confidence around vaccination [22]. Hence, review findings may also inform the use of interactive digital health interventions to combat vaccine misinformation.

### Strengths and Limitations

The strength of our study lies in its inclusive research approach, encompassing all populations, vaccine types, narratives, and digital health interventions, potentially capturing a diverse array of use cases for interactive narrative–based digital health interventions. However, several potential limitations exist with our approach. First, our reliance on peer-reviewed journal articles might lead us to overlook relevant interventions described in the gray literature. Second, our search strategy does not include the term “interaction,” which necessitates researchers’ interpretation of interactivity during title and abstract screening. Finally, this being a scoping review, we do not plan to appraise the studies for risk of bias, and our conclusions may have limitations as we are not accounting for potential biases in the included studies.

### Conclusions

In this scoping review, we will summarize the use of interactive narrative–based digital health interventions for vaccine communication. To our knowledge, this study will be the first to investigate the interactive features of these interventions and their impact on vaccine communication. Our study aims to illuminate the prevailing gaps in knowledge and provide an overview of the present research landscape. Furthermore, review findings may provide insights for public health practitioners and researchers, laying the groundwork for future studies and applications using interactive narratives for vaccine communication. Review findings may also be of relevance to vaccine communication researchers and global vaccination programs, enabling them to consider novel applications of interactive narrative–based digital health interventions in future initiatives for vaccine communication.

## Appendix

Multimedia Appendix 1 Preferred Reporting Items for Systematic reviews and Meta-Analyses extension for Scoping Reviews (PRISMA-ScR) checklist.

## Abbreviations

MeSH: Medical Subject Headings
PRISMA: Preferred Reporting Items for Systematic Reviews
PRISMA-ScR: Preferred Reporting Items for Systematic Reviews and Meta-Analyses extension for Scoping Reviews
RQ: research question
